# Validating a theory of planned behavior questionnaire for assessing changes in professional behaviors of medical students

**DOI:** 10.3389/fmed.2024.1382903

**Published:** 2024-05-14

**Authors:** Shaista Salman Guraya, Eric Clarke, Asil Sadeq, Mary Smith, Sinead Hand, Frank Doyle, Grainne Kearney, Mark Harbinson, Aine Ryan, Fiona Boland, Abdelsalam Bensaaud, Salman Yousuf Guraya, Denis W. Harkin

**Affiliations:** ^1^Institute of Learning, Mohammad Bin Rashid University, Dubai, United Arab Emirates; ^2^Centre for Professionalism in Medicine and Health Sciences, Faculty of Medicine and Health Sciences, RCSI University of Medicine and Health Sciences, Dublin, Ireland; ^3^Department of Health Psychology, School of Population Health Sciences, RCSI University of Medicine and Health Sciences, Dublin, Ireland; ^4^School of Medicine, Dentistry and Biomedical Sciences, Queens University Belfast, Belfast, United Kingdom; ^5^Data Science Centre, School of Population Health Sciences, RCSI University of Medicine and Health Sciences, Dublin, Ireland; ^6^Clinical Sciences Department, College of Medicine, University of Sharjah, Sharjah, United Arab Emirates

**Keywords:** content validity index, questionnaire validation, theory of planned behavior, undergraduate medical students, medical professionalism, professional behaviors

## Abstract

**Introduction:**

Teaching professionalism is a fundamental aspect of medical undergraduate education, delivering important domains of professional attitudes, ethics, and behaviors. The effects of educational interventions can be assessed by measuring the change in such domains, but validated assessment tools for these professionalism domains are lacking. In this study, we constructed and conducted expert validation of a modified theory of planned behavior (TPB) questionnaire to assess changes in professional behaviors (PBs) in medical students.

**Methods:**

To validate that, we modified an existing TPB questionnaire, and an 18-item questionnaire was subjected to expert panel evaluation using the content validation method. The clarity and relevance of items were assessed using a four-point rating scale (i.e., 1 = not relevant to 4 = highly relevant). Ratings of experts and free-text comments were analyzed. Quantitative evaluation of relevance and clarity was undertaken through analyses of the Item-level Content Validity Index (I-CVI) and Scale-level Content Validity Index (S-CVI). A qualitative assessment of the comments of experts was conducted to refine items, any disagreements were discussed, and a consensus decision was developed among authors for item changes.

**Results:**

Quantitative evaluation of the Item-level Content Validity Index (I-CVI) scored 0.9–1 for relevance and 0.7–1 for clarity. Qualitative evaluation resulted in (i) changes to the wording of items (e.g., choices such as “worthless/worthwhile” were replaced with “not important/important”); and (ii) suggestion of the addition of social media in the construct of subjective norms.

**Discussion:**

The proposed tool exhibits content validity and can assess TPB constructs in professionalism education. This study of content validity may help to ensure the modified TPB questionnaire accurately measures the TPB constructs, ensuring its effectiveness in accurately measuring the TPB constructs for PB in diversified educational medical institutions.

## Introduction

Medical professionalism (MP) exemplifies a set of values, beliefs, and attitudes that underpin the public’s trust in the profession of medical professionals in society (1, 2). MP is fundamental to modern medical education as it encompasses conduct, ethics, and professional identity formation, ensuring professional integrity and competence (3). Teaching MP using student-centered pedagogical approaches, such as team-based learning with real-world examples, helps bridge the gap between theory and practice (1). Professional behaviors (PBs) are the manifestations of these traits in the form of actions (4). These actions span various facets ranging from confidentiality maintenance to cultural sensitivity, ability to raise concerns, and practicing self-care, among others (1).

The aim of modern medical education is not only to impart knowledge but also to inspire and assess meaningful changes in PBs (4, 5). Published literature critiques that PB excellence is not at par with medical knowledge proficiency, highlighting areas such as MP, where improvements are needed (4). Hence, measuring changes in PBs after teaching MP is essential to assess the effectiveness of educational efforts (6). To objectively measure the impact of these educational efforts and to determine whether healthcare professionals (HCPs) can exhibit the desired changes in PB, a rigorous assessment of such MP interventions is vital. It is recommended that these interventions have a theoretical underpinning. Theory in research can help to guide intervention development, predict behaviors, and explain what works and does not work in an intervention (7). This integral process encompasses the application of underlying behavioral theories (e.g., sociological and psychological theories) and educational learning theories (e.g., constructivism theories), a careful and reflective curriculum design, and the utilization of questionnaires to gage the extent of these changes (8, 9).

Designing an effective questionnaire requires a deep understanding of the behavior change model and its constructs. Data collection questionnaires are frequently used to probe participants’ cognitive and social processes within social, behavioral, and psychological sciences (10). The theory of planned behavior (TPB) underpins the pedagogy of MP by emphasizing the interplay between TPB domains (i.e., attitudes, subjective norms, and perceived behavioral control), making them a model to understand, predict, and induce changes in PB (11). TPB is a social psychological theory, widely used to predict human intentions which are precursors of behaviors (12, 13). Despite the fact that there are two detailed guides for developing TPB questionnaires (14, 15), the published literature is critical of ambiguity and confusion in the statements of the TPB-based questionnaire, which undermine the validity and reliability of data collected (16, 17).

Essential strategies to develop a robust questionnaire involve the use of clear questions, unbiased language, and relevant content to ensure accurate responses. To do that, it must meet rigorous scientific criteria through psychometric evaluations to assure reliability and validity, answering potential objections of questionnaire-based research as “soft science” (18). This can be further achieved with piloting to intensify the robustness of results. Constructing and validating a questionnaire using theoretical underpinning is a meticulous process that ensures the instrument aligns with the chosen behavioral theories. Prior to implementing questionnaire-based interventions in predictive studies, it is imperative to ensure that the questionnaire is rigorously developed and validated.

To overcome the challenges in the validity and reliability of the TPB questionnaire, researchers must tailor its statements based on aspects of behaviors. A recent study (19) coined the Target, Action, Context, and Time (TACT) principle with the potential to mitigate challenges in questionnaire construction. While TPB and TACT principles provide guidance, the role of curriculum development and curated resource selection cannot be ignored. Thus, we aimed to validate a bespoke questionnaire, modified from Medisauskaite et al. (20), to measure the change in PB; the TPB was adopted to examine a change in intentions by looking into attitudes, subjective norms, and perceived behavioral controls of medical students (21).

## Methods

### Study design and rationale

This study is part of “PROfessionalism in Partnership for Education Research (PROPER)” research, which seeks to modify PBs of pre-clinical undergraduate medical students within the context of the hidden curriculum in undergraduate settings. This innovative approach involves the development of tailored resources dedicated to undergraduate medical students and the implementation of systematically designed workshops. These workshops aim to create safe pedagogical spaces to facilitate critical incident discussions. The PROPER intervention, comprising a series of expert-designed online workshops, will be delivered to pre-clinical undergraduate medical students, focussing on four key themes of importance to medical students and the hidden curriculum, namely, confidentiality, raising concerns, self-care/wellbeing, and cultural sensitivity. These themes were chosen after a detailed literature exploration and repeated meetings within research teams keeping the undergraduate medical students in mind.

Questionnaires are one of the most frequently utilized media within social, behavioral, and psychological sciences to access participants’ cognitive and social processes. To measure change in PBs, we modified and validated an existing TPB questionnaire (20). To ensure the robustness of our questionnaire, we conducted a comprehensive literature review to identify similar concepts. Consequently, we adapted questionnaire items from the published literature (20) to align with the specific objectives of the PROPER study.

However, to develop valid interventions based on predictive studies using questionnaire-based methods, it is vital that the questionnaire has been sufficiently developed and validated before a tool is tested in a pilot study or employed in a full research project (22).

### Content validity

Content validity has been defined as the ability of a survey to measure the intended construct accurately and comprehensively (23). To ensure that, we adopted and modified a four-step approach of judgments, opinions, and remarks of experts on a given concept by Guraya et al. (24) for conducting the process of content validity.

#### Step 1: preparation of content validation forms

We prepared a bespoke content validation form (Supplementary material), which ensured that the required task was explicitly stated, and expectations were conveyed to the review panel. The validation form included a brief introduction to the PROPER study and the rationale for using TPB constructs. It also provides definitions of TPB constructs for validating the questionnaire. The form elucidated the detailed structure of an 18-item questionnaire, which measured four constructs; the attitudes (seven items), subjective norms (three items), perceived behavioral control (five items), and intentions (three items) of participants. All items were constructed on a 7-point rating scale, where 1 represents the least likely/strongly disagree/worthless and 7 represents most likely/strongly agree/worthwhile, etc. The relevance and clarity were determined using a 4-point rating scale (1 = not relevant, 2 = somewhat relevant, 3 = quite relevant, and 4 = highly relevant) to score individual items.

#### Step 2: selection of panel experts

Assessment of the content validity can be conveniently performed through the use of a panel of content experts who individually and critically evaluate the relevance and clarity of each proposed item. There is no consensus about the required number of content experts for content validity; indeed, a range of 2–20 has been proposed in the literature (22, 23, 25, 26). A minimum of five experts is necessary to participate in a content validity study in order to generate statistically reliable outcomes. Ideally, 10 respondents should be involved in the process as it reduces the variability in responses and the likelihood of random agreement (27).

Keeping the relevancy and availability of the experts for the selected topic in mind, we purposely selected a panel of experts to participate in our study via email. To achieve this, experts were chosen based on (i) their experience and expertise in the field of medical education and MPs and (ii) who could review, analyse, and facilitate the content validity process. We searched the databases of Science Direct and Web of Science for content experts with publications in the field of MP in peer-reviewed journals across various geographical regions. Following their confirmation and agreement to participate in this research, all further correspondence with the experts was carried out by email. Upon consenting, they received the validation form as well as an elucidation of its contents.

#### Step 3: conducting content validation

In November 2022, emails were sent to the selected experts to participate in the validation process of the TPB questionnaire. An explicit set of instructions was sent to reduce any problems that participants may experience in both understanding and responding to the TPB questionnaire. Experts were requested to critically review each construct domain and its items, before providing a quantitative and qualitative evaluation. The experts assessed relevance and clarity of each item (1 = not relevant to 4 = highly relevant) and clarity (1 = unclear to 4 = very clear) on a 4-point scale. The 4-point scale was chosen because it omits the option of a neutral answer that could have a strong buffering effect on the study findings among a small pool of experts (23, 26, 27). The experts were encouraged to provide written comments to improve the relevance and clarity of items to the TPB constructs. In the case where any new items were needed to be included in each construct, experts were advised to provide new items or delete the existing ones.

#### Step 4: quantitative and qualitative evaluation

Using Microsoft Excel, we performed an analysis of the Content Validity Index (CVI). Two forms of CVI have been described in the literature, Item-level Content Validity Index (I-CVI) and Scale-level Content Validity Index (S-CVI) (23). S-CVI is calculated as the average of the I-CVI scores for all items (S-CVI/Ave). Each response from 10 experts was analyzed using two steps: For I-CVI, each item score was calculated individually, and for S-CVI/Ave, the calculation of the overall questionnaire was based on the average of individual item ratings.

We used the qualitative comments by experts to refine items in all four constructs of the TPB questionnaire. All comments were discussed among the panel, and a consensus was developed to make decisions to decide which items to change or remove.

### Analysis

According to Lynn (26), an I-CVI score of 0.78 or higher for each item was considered for inclusion in validation, particularly when conducted by nine or more experts. For the S-CVI scores, a new tool should achieve at least 0.8 or higher agreement to be considered acceptable content validity (25).

However, a common concern regarding the CVI rating process was the involvement of only 10 experts in rating survey items, which raises the risk of uniform ratings and can lead to loss of information by assessors (28). To address this methodological challenge within the CVI, we examined the potential influence of chance on the responses of expert raters to survey items (22, 28). To account for the chance agreement among raters (reliability), we calculated K*, which factors in both the probability of chance agreement and the extent of chance agreement among raters (28). This approach enhances the validity of the instrument by considering the probability of chance agreement (pc), ultimately reducing the potential for errors in data analysis. Once Pc was obtained for each item, K* was calculated using the equation presented in Box 1.

BOX 1 **Equations**

(A) Probability of chance agreement (Pc) equation:

Pc = (N!/A! [N-A]!)* 0.5 N

(B) Kappa statistic equation:

K* = (I-CVI – Pc)/(1-Pc)

N: total number of experts; A: number of experts in agreement regarding the relevance and clarity of the item;

The CVI score was then adjusted with the multi-rater K* to eliminate the possibility of an increase in value due to coincidental agreement in the assessment process of each expert (28). Evaluation criteria for K* results are categorized into: “fair” (0.40–0.59), “good” (0.60–0.74), or “excellent” (=K* > 0.74) (29).

## Results

Ten experts, comprising of medical educators and clinicians, were involved in the content validity and reliability assessment. The number of experts was determined according to Polit’s research to avoid the coincidence of arguments (22). The validity and reliability were performed during the months of November and December 2022 after obtaining informed consent from the experts.

### Quantitative assessment

The results for the relevance of the CVI assessment during the content validity process quantitatively showed that the I-CVI on all items depicted a range score between 0.9 and 1 for relevance (Table 1). Hence, all items retained their relevancy.

**Table 1:** Content validity on four domains of TPB using PROPER undergraduate medical resources.

	Relevance			Clarity		
TPB domains	*N*	I-CVI	K*	*N*	I-CVI	K*
**Attitudes** Participants’ positive and negative evaluations on confidentiality can be cognitive or affective (experience) components Belief in the impacts of behavior Evaluation of impacts of behavior						
Overall, I think maintaining patient confidentiality is worthless—worthwhile	10	1.0	0.99	10	1.0	0.99
Overall, I think maintaining patient confidentiality is unrealistic—realistic	10	1.0	0.99	9	0.9	0.88
Overall, I think maintaining patient confidentiality is unclear—clear	9	0.9	0.88	7	0.7	0.46
Overall, I think maintaining patient confidentiality is wrong thing to do—right thing to do	10	1.0	0.99	10	1.0	0.99
Overall, I think maintaining patient confidentiality is bad practice—best practice	10	1.0	0.99	10	1.0	0.99
I am more likely to speak to colleagues on confidentiality than PROPER confidentiality guidance resources for UG students	10	1.0	0.99	7	0.7	0.46
I am more likely to consult PROPER confidentiality guidance resources for UG students than institutional confidentiality guidance in practice	10	1.0	0.99	7	0.7	0.46
**Subjective norms** Participants feel that others with significant influence support or maintain their confidentiality practices Belief in others’ opinions Motivation to comply with others’ opinions						
People who are important to me think I should maintain the confidentiality of my patients	10	1.0	0.99	9	0.9	0.88
It is expected of me to maintain the confidentiality of my patients	10	1.0	0.99	9	0.9	0.88
Please indicate how much pressure you feel from each of the following organizations or people to use the PROPER confidentiality guidance resources for UG students My college/institution Myself/My trust Medical Council Personal tutor/supervisor Peers Teachers Patients Society The media	10	1.0	0.99	8	0.8	0.71
**Perceived behavioral control** Participants’ perceptions that they have self-control in maintaining confidentiality practices by using PROPER confidentiality guidance resources Belief in having behavioral control Power perceived in facing obstacles						
Overall, I think maintaining patient confidentiality is difficult—easy	10	1.0	0.99	10	1.0	0.99
I am confident that I cannot apply PROPER confidentiality guidance resources for UG students	10	1.0	0.99	9	0.9	0.88
I have enough time to refer to PROPER confidentiality guidance resources for UG students	9	0.9	0.88	9	0.9	0.88
I can easily navigate the PROPER confidentiality guidance resources for UG students to check confidentiality guidelines	10	1.0	0.99	10	1.0	0.99
For me to apply PROPER confidentiality guidance resources for UG students in practice is easy—difficult	10	1.0	0.99	8	0.8	0.71
**Intentions** How likely a participant is to do or engage in maintaining confidentiality practices by using PROPER confidentiality guidance resources State of mind in the form of commitment to an action						
I intend to refer to PROPER confidentiality guidance resources for UG students the next time I am uncertain	10	1.0	0.99	9	0.9	0.88
I want to use the PROPER confidentiality guidance resources for UG students	10	1.0	0.99	9	0.9	0.88
I do not plan to use the PROPER confidentiality guidance resources for UG students	10	1.0	0.99	9	0.9	0.88
S-CVA Ave	1.0			0.9		

N, Number of experts in agreement; K*, kappa statistic; I-CVI, Item-level Content Validity Index; S-CVI/Ave, Scale-level Content Validity Index Average; UG, Undergraduate; PROPER, PROfessionalism Partnership for Education Research; UG: undergraduate.

With regard to clarity, the I-CVI score ranged between 0.7 and 1. Three items in the attitude construct scored 0.7 on the clarity scale and k* = 0.46, thus falling in the fair category and requiring qualitative improvement in terms of clarity.

For the S-CVI/Ave, based on I-CVI for relevance and clarity, a score of 1.0 and 0.9 was recorded, respectively. Henceforth, we inferred that I-CVI and S-CVI/Ave achieved a satisfactory level and our TPB questionnaire scale achieved an optimal level of content validity (25).

### Qualitative assessment

The pseudonymised qualitative comments and inputs to the statements suggested “*choice of phrase*”, “*reference to the context*”, “*distinct separation of personal and professional self*”, and an additional source of subjective norms; “the social media.” These were undertaken to improve the clarity of the questionnaire (Table 2).

**Table 2:** Pre-post validated items forms for the improvement of item clarity.

Theme	Pre-validated item form	Post-validated item form
Choice of phrase and reference to the context	Overall, I think maintaining patient confidentiality is worthless—worthwhile	Overall, I think maintaining patient confidentiality in daily medical student clinical placement is unimportant—extremely important
Reference to the context	Overall, I think maintaining patient confidentiality is unclear—clear	Overall, I think maintaining patient confidentiality in daily medical student clinical placement is unimportant—extremely important
Distinct separation of personal and professional self	People who are important to me think I should maintain the confidentiality of my patients	People who are important in my *personal capacity* think I should maintain the confidentiality of my patients People who are important in my *professional capacity* think I should maintain the confidentiality of my patients
A new source of subjective norm	Please indicate how much pressure you feel from each of the following organizations or people to use the PROPER confidentiality guidance resources for UG students My college/institution Myself Medical Council Personal tutor/supervisor Peers Teachers Society Patients The media	Please indicate how much pressure you feel from each of the following organizations or people to use the PROPER confidentiality guidance resources for UG students My college/institution Myself Medical Council Personal tutor/supervisor Peers Teachers Society Patients The media/*social media*

#### Choice of phrase

Some items were refined for clarity. For example, a consensus discussion was undertaken by experts to replace “worthless/worthwhile” with “unimportant/important” and “bad practice/best practice” with “unprofessional/professional.”

*“How did you arrive to ‘worthless – worthwhile?’ Could it also be, e.g., not at all important – exceptionally important, the word worthless seems a bit out of place to me” [E6]*

*“Does this question relate to best professional practice?” [E9]*

*“Regarding ‘bad practice… is about—best practice’, Should it be unprofessional vs. professional?” [E6]*

#### Reference to context

Experts highlighted that the use of “realistic/unrealistic” and “wrong thing to do/right thing to do” can be confusing if not put into an explicit context. Consequently, the wording was appropriately phrased in the revised questionnaire in the context of “daily clinical practice.”

*“Unrealistic/realistic to whom?—This depends on context. Would students need a box/space where they can” [E2]*

*“Realistic’ may need to be slightly more unpacked or perhaps put in context, e.g., in daily medical student clinical placement” [E3]*

*“This again will depend on context so students may find it difficult to answer and you may wish to know in what context they do respond.” [E6]*

Another item in the attitude construct scored less on clarity (I-CVI of 0.7) and the rewording of the item deemed fit considering K* = 0.46 for relevance. Again, providing context for ‘daily clinical practice’ clarified the item for the readers.

*“I am not quite sure if this question is about how to maintain confidentiality, I would suggest a minor rephrasing: ‘Overall, I think how to maintain confidentiality is clear -unclear’” [E9]*

*“Do you mean that the meaning of confidentiality is not clear? I.e., what can and can they not share and to whom?” [E6]*

*“I think ‘unclear’ needs to be specified-does this mean the concept itself is poorly defined or unclear” [E3]*

*“This item is relevant, but the sentence is unclear to me. What do you mean by maintaining confidentiality is unclear?” [E11]*

#### Distinct separation of personal and professional self

In the subject norms construct, experts highlighted the extreme relevance of distinct influence exerted by personal and professional self while maintaining confidentiality in daily clinical practice. Hence, there was an addition of an item in the relevant construct.

“*People important to me—is that in a personal or professional sense?*”

*“Consider separating self and trust” [E8]*

#### The social media

In the subjective norms construct, experts suggested a few more sources that influence the motivation to use PROPER resources for confidentiality maintenance, for example, two experts commented:

“*Do you mean social media?” [E5]*

## Discussion

This paper sought to present the methodological process of modifying and validating a TPB questionnaire (through quantitative and qualitative approaches) to allow valid assessment of specific MP domains covered in an educational intervention, in our case the PROPER study. The PROPER intervention, comprising a series of expert-designed online workshops for pre-clinical undergraduate medical students, plans to focus on four key domains of importance to medical students and the hidden curriculum, namely, confidentiality, raising concerns, self-care/wellbeing, and cultural sensitivity. Importantly, the methodology used in this study could be adapted by educators to assess the impact of educational interventions in a number of other domains. Clarity and relevance are closely intertwined with these concerns and their analysis can help ensure that our items are explicit, contextually accurate, and intricately aligned with the construct of interest (23). The I-CVI scores for relevance ranged from 0.9 to 1, signifying that all items were considered highly relevant by the panelists (25). The I-CVI calculated for each item within the questionnaire was pertinent to the construct being measured (23). While clarity items ranged between 0.7 and 1, indicating areas for development (25). Three items within the attitude construct indicated a fair clarity (I-CVI of 0.7) (26). However, Brod et al. (30) recommend that a minimum score of I-CVI should be ≥0.78 if the number of panelists is six or more. Despite the S-CVI/Ave for clarity was 0.9, signaling that most items were rated as clear, there was room for improvement for items in the attitudes construct. This suggests that, in general, the items effectively measure the intended constructs and are well accepted by expert panelists in terms of relevance and clarity (25, 27, 31).

Clarity is vital for ensuring the quality and validity of research findings and for accurately measuring the factors that influence behavioral intentions (32). To further assess the degree of agreement among panelists regarding clarity, the kappa statistics for these items indicated “fair” agreement (29). This highlighted the concerns related to clarity pointing to ambiguity and confusion in the constructed questionnaire for the PROPER study. Such a finding is expected in questionnaire development, as some constructs may inherently involve complex or multifaceted concepts that are difficult to express clearly and explicitly (32).

A thorough qualitative feedback provided a deeper understanding of the questionnaire items and identified potential areas for improvement in terms of clarity. Regarding the construct of subjective norms, experts rightly pointed out how diverse social and cultural settings, context, the rising importance of social media, and the concept of personal and professional identity influence individuals’ subjectivity and decision-making (33). Recognizing this issue, we addressed the clarity of these items to ensure that potential PROPER research participants could accurately and uniformly interpret the statements. The “*choice of phrase*” and “*reference to the context*” highlight the need for precise and context-specific wording in questionnaire items. In addition, the suggestion to create a “*distinct separation of personal and professional self*” resonates with the complexities of TPB constructs, particularly in healthcare and medical contexts. This highlights the nuanced role of subjective norms in personal and professional identity formation (34). This differentiation is fundamental in healthcare, especially when novice HCPs on the outskirts of professional communities of practices splint and patch their fledgling professional identities within the context of their personal roles (35). Moreover, the proposal to include “*social media*” as an additional source of subjective norms recognizes the evolving social landscape and the impact of digital communication on individuals’ behaviors and attitudes (34). This suggests considering the dynamic change of social influence and how they can be incorporated into our TPB-based questionnaires, which can lead to the specification of social media in the subjective norms construct. Such ambiguous statements were clearly separated to avoid indefinite boundaries.

To enhance the predictive power of TPB-based questionnaires, questions should be behavior-specific and situated within a particular context. This aspect was well grounded by the panelists. Using the TACT principle (19), it is imperative to construct questionnaire items to ensure situational context, which could enable participants to relate to the significance of PB. By acknowledging a diverse range of contextual expertise of the panelists, we contextualized the domain of maintaining confidentiality in the “daily placement of a medical student.” Underpinning the TACT principle (15), we improved our TPB questionnaire which aims to *target* undergraduate medical students to measure their change in PB by *applying* confidentiality principles in their daily context of medical placements in a pre–post and delayed post *time frame.*

A wealth of published literature on TPB has discussed that the subjective norm construct is a less effective predictor compared to others such as attitude and perceived behavioral control for PB in the healthcare context (16, 17). This may be particularly due to the problem participants encountered with subjective norm items. These items assess the degree to which an individual is inclined to align his actions with the perceived expectations. To address this, we respected the normative beliefs of our panelists and brought a sharp distinction between personal and professional self. This supported the advantages of capturing the normative beliefs of our PROPER study participants if they hold a personal or professional self-significance in their professional practice.

## Strength and limitations

The applied methodology that is tailored to the PROPER study is theory based with a robust validation process consisting of diverse panelists. However, a diverse sample of experts with a varied understanding of TPB may have led to individual interpretations and inherent subjectivity while validating the items. Second, the absence of a pilot test with a representative sample could have refined the clarity and relevance of statements and enhanced the predictive validity of the questionnaire of the PROPER study (22, 23). However, a robust quantitative and qualitative process of validation underlines the strength of our modified questionnaire.

## Conclusion

This article details the methodological process conducted to validate a modified TPB-based questionnaire with the goal of ensuring its efficacy in accurately measuring TPB constructs. This process may be able to measure changes in PBs across a range of MP-based educational interventions in diverse medical institutions. By integrating qualitative insights and highlighting its clarity and relevance, this validated questionnaire may enable accurate measurement of TPB constructs in the PROPER study. The utilization of such practices may benefit medical students by identifying the facilitators and barriers to PBs and may also improve patient care and experiences through targeted educational interventions.

## Data availability statement

The original contributions presented in the study are included in the article/Supplementary material, further inquiries can be directed to the corresponding author.

## Ethics statement

The studies involving humans were approved by Board Royal College of Surgeons Ireland Dublin (REC202205007) and Queens’ University Belfast (MHLS 22_184). The studies were conducted in accordance with the local legislation and institutional requirements. The participants provided their written informed consent to participate in this study.

## Author contributions

SSG: Conceptualization, Data curation, Formal analysis, Investigation, Methodology, Project administration, Visualization, Writing – original draft, Writing – review & editing. EC: Data curation, Investigation, Project administration, Resources, Visualization, Writing – review & editing. AS: Methodology, Resources, Validation, Visualization, Writing – review & editing. MS: Formal analysis, Project administration, Resources, Visualization, Writing – review & editing. SH: Formal analysis, Project administration, Resources, Visualization, Writing – review & editing. FD: Investigation, Methodology, Project administration, Resources, Supervision, Validation, Visualization, Writing – review & editing. GK: Investigation, Methodology, Project administration, Resources, Validation, Visualization, Writing – review & editing. MH: Investigation, Methodology, Project administration, Resources, Visualization, Writing – review & editing. AR: Investigation, Project administration, Resources, Visualization, Writing – review & editing. FB: Investigation, Methodology, Project administration, Resources, Visualization, Writing – review & editing. AB: Investigation, Project administration, Resources, Validation, Visualization, Writing – review & editing. SYG: Investigation, Methodology, Project administration, Resources, Supervision, Validation, Visualization, Writing – review & editing. DH: Funding acquisition, Investigation, Methodology, Project administration, Resources, Supervision, Validation, Visualization, Writing – review & editing.
