# Supplementary material for: Validating a theory of planned behavior questionnaire for assessing changes in professional behaviors of medical students
Source: Front Med (Lausanne). 2024 May 14;11:1382903. doi: 10.3389/fmed.2024.1382903 (PMC11130381; doi:10.3389/fmed.2024.1382903)
Supplement: Supplementary file 1 [file Table_1.DOCX]

**EXPERT REVIEW AND EVALUATION FORM** **FOR TPB Questionnaire (Confidentiality Sub-domain)**

| Expert Name | : |  |
| --- | --- | --- |
| Email | : |  |
| Institution | : |  |

**INTRODUCTION:**

We are conducting a PROPER study on undergraduate medical students of RCSI Dublin and QUB. We are interested in determining the impact of specially curated workshop (Confidentiality sub-domain), in changing professional behaviours of our participants using a pre-post questionnaire. Questionnaires are one of the most frequently utilized media within social, behavioural and psychological sciences to access participants’ cognitive and social processes. To develop valid interventions based on predictive studies using questionnaire-based methods, it is vital that the questionnaire has been sufficiently developed.  To develop this questionnaire, we employed the Theory of Planned Behavior (TPB) which examines a change in intentions by looking into attitudes, subjective norms, and perceived behavioral controls. TPB is a social psychological theory which has been widely used to predict human intentions which are precursor of behaviors.

This 26-item questionnaire was constructed by using [TPB Manual](https://drive.google.com/file/d/1sRTaS4qENqKgEJH-X61jwLZHcVvfT7c3/view?usp=share_link). The questionnaire will measure 4 concepts; the attitudes (8 items), subjective norms (11 items), perceived behavioural control (4 items) and intentions (3 items) (precursor to behaviour change) of participants and compare them to the control group of the same institution. All items are constructed on a rating scale of 7 where 1 represents the least likely, strongly disagree, worthless and 7 represents most likely, strongly agree and worthwhile etc. It is important to reduce any problems that participants may experience in both understanding and responding to TPB questionnaire. In case that any new item needs to be included in each concept category, we have provided an empty row. You may add more rows if needed. Your opinion as an expert is sought to validate this questionnaire in terms of relevance and clarity. We appreciate your time and expertise.

**INSTRUCTIONS:**

Please **RATE (relevance and clarity both) and** **WRITE** your review in the Expert Review form to validate the TPB Questionnaire (Confidentiality Sub-domain).

| **Overarching concept** | **Concept definition** | **Specific domains** | **Statement** | **RELEVANCE RATING**  **1 = the item is not relevant to the concept**  **2 = the item is somewhat relevant to the concept**  **3 = the item is relevant to the concept**  **4 = the item is very relevant to the concept** | **CLARITY RATING**  **1 = the item is unclear**  **2 = the item is rather unclear (item needs revision)**  **3 = the item is quite clear (item is clear but needs minor revision)**  **4 = very clear (item is understandable)** | **EXPERT REVIEW** |
| --- | --- | --- | --- | --- | --- | --- |
| **Attitudes** | Participants positive and negative evaluation on confidentiality, can be cognitive or affective (experience) components | -Belief in impacts of behaviour  - Evaluation on impacts of behaviour | 1.1. Overall, I think maintaining patient confidentiality is worthless – worthwhile |  |  |  |
|  |  |  | 1.2. Overall, I think maintaining patient confidentiality is difficult - easy |  |  |  |
|  |  |  | 1.3. Overall, I think maintaining patient confidentiality is unrealistic – realistic |  |  |  |
|  |  |  | 1.4. Overall, I think maintaining patient confidentiality is unclear - clear |  |  |  |
|  |  |  | 1.5. Overall, I think maintaining patient confidentiality is wrong thing to do – right thing to do |  |  |  |
|  |  |  | 1.6. Overall, I think maintaining patient confidentiality is bad practice – best practice |  |  |  |
|  |  |  | 2.3. I am more likely to speak to colleagues on confidentiality than PROPER confidentiality guidance resources for UG students (R) |  |  |  |
|  |  |  | 2.4. I am more likely to consult PROPER confidentiality guidance resources for UG students than institutional confidentiality guidance in practice (R) |  |  |  |
|  |  |  | The scales used below will impact the questions asked. |  |  |  |
| **Subjective Norms** | Participants feeling that others with significant influence support their maintain confidentiality practices | - Belief in others’ opinions  - Motivation to comply with others’ opinions | 2.1. People who are important to me think I should maintain the confidentiality of my patients |  |  |  |
|  |  |  | 2.2. It is expected of me to maintain the confidentiality of my patients |  |  |  |
|  |  |  | 3. Please indicate how much pressure you feel from each of the following organisations or people to use the PROPER confidentiality guidance resources for UG students |  |  |  |
|  |  |  | - 3.1. My college/institution |  |  |  |
|  |  |  | - 3.2. Myself/My trust |  |  |  |
|  |  |  | - 3.3. Medical council |  |  |  |
|  |  |  | - 3.4. Personal tutor/supervisor |  |  |  |
|  |  |  | - 3.5. Peers |  |  |  |
|  |  |  | - 3.6. Teachers |  |  |  |
|  |  |  | - 3.7. Patients |  |  |  |
|  |  |  | - 3.8. Society |  |  |  |
|  |  |  | - 3.9. The media |  |  |  |
|  |  |  |  |  |  |  |
| **Perceived Behavioural Control** | Participants perceptions that they have self-control in maintaining confidentiality practices by using PROPER confidentiality guidance resources | - Belief in having behavioural control  - Power perceived in facing obstacles | 2.5. I am confident that I cannot apply PROPER confidentiality guidance resources for UG students |  |  |  |
|  |  |  | 2.6. I have enough time to refer to PROPER confidentiality guidance resources for UG students |  |  |  |
|  |  |  | 2.7. I can easily navigate the PROPER confidentiality guidance resources for UG students to check confidentiality guidelines |  |  |  |
|  |  |  | 4. For me to apply PROPER confidentiality guidance resources for UG students in practice is easy - difficult |  |  |  |
|  |  |  |  |  |  |  |
| **Intentions** | How like a participant is to do or engage in maintaining confidentiality practices by using PROPER confidentiality guidance resources | - State of mind in the form of commitment to action | 2.8. I intend to refer to PROPER confidentiality guidance resources for UG students the next time I am uncertain |  |  |  |
|  |  |  | 2.9. I want to use the PROPER confidentiality guidance resources for UG students |  |  |  |
|  |  |  | 2.10. I don’t plan to use the PROPER confidentiality guidance resources for UG students (R) |  |  |  |
|  |  |  |  |  |  |  |
